# Temporal evolution of anemia in children aged six to 59 months in the state of Pernambuco, Brazil, 1997 to 2016

**DOI:** 10.1590/1980-549720230023.2

**Published:** 2023-05-08

**Authors:** Maríllia Raquel de Lima, Maria de Fátima Costa Caminha, Suzana Lins da Silva, Juliana de Castro Nunes Pereira, Déborah Lemos Freitas, Pedro Israel Cabral de Lira, Malaquias Batista

**Affiliations:** Instituto de Medicina Integral Professor Fernando Figueira – Recife (PE), Brasil.; Universidade Federal de Pernambuco, Departamento de Nutrição – Recife (PE), Brasil.

**Keywords:** Anemia, Child, Nutritional status, Health surveys, Anemia, Criança, Estado nutricional, Inquéritos epidemiológicos

## Abstract

**Objective:**

To analyze the temporal evolution of anemia in children aged six to 59 months in Pernambuco, based on population surveys from 1997, 2006, and 2016 and the factors associated with the situation in 2016.

**Methods:**

The field studies took place in the participants’ households, in the Recife Metropolitan Area, as well as in the urban and rural inland. The trend study of anemia in children used data from the State Health and Nutrition Survey (*Pesquisa Estadual de Saúde e Nutrição* — PESN) II (40.9%) and III (32.8%). Data from PESN IV were collected using questionnaires administered to families to verify socioeconomic and individual conditions, as well as anthropometric — weight and height — and biochemical — hemoglobin — records. We adopted the test for trend in proportion for the time trend study and Poisson regression for hypothesis tests for the associated factors. Statistically significance was set at a p-value<0.05.

**Results:**

In 2016, the prevalence of anemia was 24.2%, indicating a significant reduction in disease incidence. In children aged 6–23 months, this number decreased from PENS II and III to PENS IV — 63 and 55.6 to 37.7% (p<0.001), respectively. In 2016, the statistically significant variables for anemia in children were maternal hemoglobin, child's age, current or recent case of diarrhea, and weight-for-age index

**Conclusion:**

Between 1997 and 2016, anemia rates decreased, showing an epidemiological trend that can contribute to continuously improve the health of children under five years of age in Pernambuco.

## RESUMO

**Objetivo:**

Analisar a evolução temporal da anemia em crianças de seis a 59 meses em Pernambuco, com base nos inquéritos populacionais de 1997, 2006 e 2016 e os fatores associados à situação em 2016.

**Métodos:**

Os estudos de campo ocorreram nos domicílios dos participantes, na Região Metropolitana do Recife, interior urbano e rural. No estudo de tendência da anemia em crianças, utilizaram-se dados da II (40,9%) e III PESNs (Pesquisa Estadual de Saúde e Nutrição) (32,8%). Os dados da IV PESN foram coletados por formulários com famílias para verificação das condições socioeconomicas e individuais, bem como registros antropométricos, peso e altura, e bioquímicos, hemoblobina. Para o estudo de tendência temporal, utilizou-se o teste de tendência de proporção; e para os fatores associados a regressão de Poisson para testes de hipóteses. Estatisticamente considerou-se significante o valor p<0,05.

**Resultados:**

A prevalência de anemia, em 2016, foi de 24,2%, expressando uma diminuição significativa na ocorrência da doença. Nas crianças de 6–23 m., houve redução da II e III para IV PESN de 63 e 55,6 para 37,7% (p<0,001), respectivamente. Em 2016, as variáveis com significância estatística para a anemia em crianças foram a hemoglobina materna, a idade da criança, a ocorrência atual ou recente de diarreia e o índice P/I.

**Conclusão:**

Entre 1997 e 2016, houve redução da anemia, demostrando uma tendência epidemiológica que pode contribuir para melhoria contínua da saúde das crianças abaixo de cinco anos em Pernambuco.

## INTRODUCTION

Population-based studies of regions and countries are historically recent, accepting as consensual evidence that anemias, as well as deficiencies of vitamins A, D and of iodine, have been the dominant micronutrient deficiencies for several decades^1^, recognized as international collective health priorities and mobilizing research facilities and public interventions aimed at their reduction and possible epidemiological control worldwide.

In the specific case of anemias, given their magnitude and consequences — direct or associated with other comorbidities —, the World Health Organization (WHO) classified them, in 2011, as a serious, permanent, and rising problem in some restricted national or international cases, estimating that one third or at least a quarter of the entire human population had mild, moderate, and severe circulating hemoglobin deficiencies,^2^ which characterize, from a laboratory perspective, the elective indicator of a specific deficiency^1^, mainly due to qualitative and quantitative restrictions of iron in the diet^3^.

In 2011, anemia was classified as a moderate to severe public health problem among women of reproductive age and children under five years in most WHO member states^2,4,5^. Since 2000, the global prevalence of anemia in children has slowly decreased over the years — from 48% to 39.8% —, and as of 2010, it has been stagnant^6^.

As a biological principle, human susceptibility to the problem is universal, and thus, anemia can occur at any life stage, from fetal to old age^7^. However, on an epidemiological scale, the problem is more relevant in the maternal-child segment, that is, women of reproductive age, especially during pregnancy, and children under five years^1,2^.

In this group, a set of common and interactive factors stands out in the triad reproductive period/pregnancy/growth and in the development of children in the first months and years of life, favoring the occurrence of anemia: on the one hand, pathophysiological processes and on the other, concomitant adverse socio-environmental factors^8^, which can be grouped into the so-called “poverty ecosystem”, a very common context in backward or developing countries^1^.

“Poverty ecosystems” cannot be defined as a single model but basically include low household income, unhealthy living and surrounding conditions, low schooling, qualitative and quantitative restrictions of health actions, and limited social services support network^9^.

Studies show that iron deficiency is the most common cause of anemia worldwide. Nevertheless, acute and chronic inflammation, parasitic infestations, and hereditary or acquired diseases that affect the synthesis, production, or survival of red blood cells can cause anemia^10^.

The current study, research focused on time trends, could follow two directions. The first has a “spontaneous” nature and mainly involves the course of changes that happened in the significant process of nutritional transition, very characteristic of the last decades of the previous century^11^. Although marked by the transition from malnutrition to overweight/obesity, this stage also shifts the temporal course of specific nutritional deficiencies, such as anemias^12^.

Precisely for being an aspect that is little valued in conventional studies, we chose to address the possible temporal shifts that could be affecting anemia in the most vulnerable group of children, that is, those under five years in a geographical area recognized as poor^13^.

The other, more interventionist than spontaneous, component would consider possible trends in objectives and goals of the millennium, such as the adherence of governments and international institutions to new and necessary panels in the context of policies and programs for public or governmental interventions^14^. In reality, a favorable or desired expectation is created to improve the incidence/prevalence levels of problems such as nutritional anemias in higher-risk groups, as is the case of children and women of reproductive age, especially during pregnancy.

Based on these two “axial” foci, we contemplated analyzing possible prospective trends since 1997 in the state of Pernambuco. An additional argument is the great relevance of hemoglobin deficiency in children, which produces very specific adverse consequences for this critical biological transition, leading to changes in neurotransmission and myelination of nerve fibers^15^, with implications for neuropsychomotor development and its impacts on learning, social performance, and ideo-affective processes, increasingly required for human development^16,17^.

As the only state to have carried out periodic health and nutrition surveys of the population since 1992, with a special focus on the maternal-child domain, Pernambuco is very well suited to meet the objectives of temporal assessments.

Thus, this study aimed to analyze the temporal evolution of anemia in children aged six to 59 months in Pernambuco based on population surveys from 1997, 2006, and 2016 and the factors associated with the situation in 2016.

## METHODS

This is a cross-sectional study with a database scope and an observational, descriptive, and analytical design. We used secondary data extracted from databases — collected at the Public Health Laboratory of Universidade Federal de Pernambuco (UFPE) — of the State Health and Nutrition Survey (*Pesquisa Estadual de Saúde e Nutrição* — PESN) II, III, and IV, performed in Pernambuco in 1997, 2006, and 2016, respectively.

The study population comprised children aged six to 59 months of both sexes, living in Pernambuco in 1997, 2006, and 2016, that is, covering almost 20 years. The first two investigations (1997 and 2006) used prevalence rates of anemia already published in scientific articles — 40.9% in a sample of 777 children for the 1997 PESN^18^ and 32.8% in a sample of 1,403 children for the 2006 PESN^19^. In the 2016 PESN, the population consisted of 880 children belonging to the original research database; however, this study analyzed the 727 children whose hemoglobin levels were provided. The reduced size of the study population, compared to PESN III, is due to the unilateral decrease in institutions that funded the resources available for the performance of PESN IV.

The anthropometric evaluation was carried out at the interview by previously trained team members. The measures taken met the WHO recommendations^20^. They were obtained in two stages, based on the following procedures: children under two years were weighed with their mother or guardian and with minimal clothing, in a digital scale (Tanita model – BF-683W/UM028 3601), with a capacity of 150 kg and accuracy of 100 g. Next, the mother or guardian was weighed alone to calculate the difference and record the child's final weight. The weight of children older than two years and mothers was obtained using the same scale, with the individual barefoot and wearing minimal clothing.

Children up to two years were measured (length) in the supine position, using a wooden infantometer, with a range of 100 cm and an accuracy of 0.1 cm. The height of children older than two years was determined by a portable stadiometer (Alturaexact Ltda.) — measured in millimeters, with an accuracy of up to 1 mm throughout its length. The children were placed in the upright position, barefoot, with upper limbs hanging along the body, and the heels, back, and head touching the wooden bar.

Hemoglobin levels were obtained from a capillary blood sample. Hemoglobin was determined using Urit-12 equipment (Medical Electronic Co., Ltd.), with immediate reading. The anemia diagnosis was established based on criteria recommended by the WHO, which considers anemic those children with hemoglobin below 11 g/dL^1^.

Nutritional status was assessed using the following indices based on anthropometric data: weight-for-age (W/A), length/height-for-age (L/A or H/A), and weight-for-length/height (W/L or W/H). The reference standard used to compare the measurements of weight, length/height, and body mass index (BMI) was that recommended by the WHO (Anthro – 2007)^21^, complying with the following criteria for the indices W/A, W/L, W/H, and BMI/A: underweight z-score (ZS) <-2; nutritional risk ZS ≥-2 to <-1; nutritional adequacy ZS ≥-1 to ≤1; overweight ZS >1 to <2; and obesity ZS ≥2. The criteria for L/A or H/A indices were: stunted ZS <-2 and normal length/height ZS ≥-2.

The interviews were conducted with the person responsible for the child. In their absence, the interviewer returned up to two times to complete the questionnaire.

The study database produced an ad hoc file and was constructed based on information from Pernambuco surveys in 2016. When necessary, the variables were recoded for statistical analysis according to the proposed objectives and the methodological procedures used.

Since all variables involved in the statistical analysis were categorical or categorized, they were summarized as absolute and relative frequencies. Statistical analyses were performed in the Stata 12.1 software.

We used Poisson regression with robust variance to investigate whether the occurrence of anemia in children aged six to 59 months could be associated with the various independent variables studied. Initially, a bivariate analysis was performed by adjusting simple Poisson regression models to statistically test these associations (using the Wald test) and estimate the crude prevalence ratios (PR) with their respective 95% confidence intervals (95%CI). The final multivariate model derived from the initial model after applying the backward method, set at a 0.05 significance level.

The temporal evolution of anemia in children was verified by the test for trend in proportions, and variations and their respective 95%CI were estimated among the three PESN editions. Values were obtained by the Z test.

The Ethics Committee of Instituto de Medicina Integral Professor Fernando Figueira (Imip) approved this time-trend study under the Certificate of Presentation for Ethical Consideration (*Certificado de Apresentação de Apreciação Ética* — CAAE) No. 26433219.8.0000.5201, on December 12, 2019.

## RESULTS

The prevalence of anemia in PESN IV (2016) among children under five years in Pernambuco was 24.2% (95%CI 20.3–28.5%) — prevalence of 22.5% (95%CI 18.1–27.6%) in the urban inland and the Recife Metropolitan Area (RMA) and of 28.0% (95%CI 21.0–36.4%) in the rural inland, with no statistical difference when comparing both geographic strata: p=0.231.

The mean age of the children studied was 31.5 months (standard deviation, SD=15.3) — 30.8 months (SD=15.4) in the urban area and RMA and 33.4 months (SD=15.0) in rural areas. Male children were predominant in the urban area (54.7%), while rural areas had more females (55.0%), p=0.016.

For the historical series, the sequence of results related to the prevalence of anemia in children aged six to 59 months ranged between 40.9% (1997) and 24.2% (2016). At the midpoint of the timeline, that is, PESN III, the value found was 32.8%. The statistical analysis of the time series, constructed with data from three population-based surveys, adopted the test for trend in proportions corresponding to p<0.001, thus considered significant (95%CI).

We found a downward trend in the prevalence of anemia among children in Pernambuco — a 40% relative reduction.

Table 1 presents the PR of maternal sociodemographic, obstetric, and laboratory variables, revealing a statistically significant relationship with maternal age under 20 years (p=0.05) and maternal hemoglobin levels, defined at the cut-off point <12 g/dL.

**Table 1:** Crude prevalence ratios of the association of anemia with sociodemographic, obstetric, and laboratory variables of mothers of children aged six to 59 months. State of Pernambuco, 2016. Recife (PE), Brazil.

Variable	Sample	Anemia	PR (95%CI)	p-value
	n	n (%)		
Maternal age (years) (n=682)				0.010^*^
<20	70	25 (35.7)	1.67 (1.05-2.64)	0.029
20 to 35	486	486 135 (27.8)	1.30 (0.90-1.87)	0.162
≥36	126	27 (21.4)	1.0 (Ref.)	
Maternal schooling (n=680)				0.234^*^
Illiterate/incomplete elementary school	89	28 (31.5)	1.35 (0.89-2.05)	0.156
Complete elementary school/incomplete middle school	180	55 (30.6)	1.31 (0.92-1.88)	0.136
Complete middle school/incomplete high school	159	37 (23.3)	1.0 (Ref.)	
Complete high school and over	252	67 (26.6)	1.14 (0.81-1.62)	0.455
Geographic area (n=726)				
Urban inland and RMA	506	137 (27.1)	1.0 (Ref.)	0.956
Rural inland	220	60 (27.3)	1.01 (0.78-1.31)	
*Per capita* income (MW) (n=712)				0.127^*^
<0.5	617	171 (27.7)	3.88 (0.58-25.79)	0.161
≥0.5 and <1.0	81	20 (24.7)	3.46 (0.50-23.76)	0.207
≥1.0	14	1 (7.1)	1.0 (Ref.)	
Number of household residents (n=727)				0.201^*^
1 to 3	183	45 (24.6)	1.0 (Ref.)	
4	211	57 (27.0)	1.10 (0.78-1.54)	0.585
5	138	34 (24.6)	1.00 (0.68-1.48)	0.992
6 to 16	195	61 (31.3)	1.27 (0.92-1.77)	0.151
Prenatal care (n=721)				
Yes	706	189 (26.8)	1.0 (Ref.)	0.993
No	15	4 (26.7)	1.00 (0.43-2.33)	
Place of birth (n=725)			
Public hospital	625	169 (27.0)	1.10 (0.76-1.60)	0.601
Private hospital	98	24 (24.5)	1.0 (Ref.)	
Type of delivery (n=725)				
Vaginal/natural	366	100 (27.3)	1.0 (Ref.)	0.794
Cesarean	359	95 (26.5)	0.97 (0.76-1.23)	
Maternal Hb (n=631)				
<12 g/dL	140	49 (35.0)	1.35 (1.03-1.77)	0.029
≥12 g/dL	491	127 (25.9)	1.0 (Ref.)	

* Linear trend test. PR: prevalence ratio; 95%CI: 95% confidence interval; RMA: Recife Metropolitan Area; MW: minimum wage; Hb: hemoglobin.

When considering the variables of children diagnosed with anemia (Table 2), most risk factors were statistically associated with anemia, such as the child's age (p<0.001), diarrhea in the previous two weeks (p=0.004), and indices in the W/A (p=0.048) and L/A or H/A (p=0.045) ratios.

**Table 2:** Crude prevalence ratios of the association of anemia with biological and clinical variables and anthropometric indices of children aged six to 59 months. State of Pernambuco, 2016. Recife (PE), Brazil.

Variable	Sample	Anemia	PR (95%CI)	p-value
	n	n (%)		
Age (months) (n=727)				
6 to 23	260	98 (37.7)	1.78 (1.41-2.25)	<0.001
24 to 60	467	99 (21.2)	1.0 (Ref.)	
Child's sex (n=727)				
Male	376	105 (27.9)	1.07 (0.84-1.35)	0.604
Female	351	92 (26.2)	1.0 (Ref.)	
Diarrhea in the previous two weeks (n=725)				
Yes	122	45 (36.9)	1.48 (1.13-1.94)	0.004
No	602	150 (24.9)	1.0 (Ref.)	
Cough (n=725)				
Yes	381	103 (27.0)	1.01 (0.79-1.29)	0.930
No	344	92 (26.7)	1.0 (Ref.)	
Hospitalization in the previous 12 months (n=725)				
Yes	92	29 (31.5)	1.20 (0.87-1.67)	0.272
No	633	166 (26.2)	1.0 (Ref.)	
Weight-for-age (n=719)				0.048
Underweight/nutritional risk	86	23 (26.7)	1.34 (0.85-2.11)	0.212
Nutritional adequacy	453	136 (30.0)	1.50 (1.09-2.08)	0.014
Overweight/obesity	180	36 (20.0)	1.0 (Ref.)	
Weight-for-length/height (n=709)				0.404
Underweight/nutritional risk	59	14 (23.7)	0.80 (0.49-1.31)	0.370
Nutritional adequacy	381	97 (25.5)	0.86 (0.67-1.10)	0.226
Overweight/obesity	269	80 (29.7)	1.0 (Ref.)	
Length/height-for-age (n=709)				
Stunted	62	23 (37.1)	1.43 (1.01-2.03)	0.045
Normal length/height	647	168 (26.0)	1.0 (Ref.)	

PR: prevalence ratio; 95%CI: 95% confidence interval.

We obtained the adjusted prevalence rates from the multivariate analysis (Table 3), selecting all variables with p<0.20 in the bivariate analysis to build the initial multivariate regression model. The final multivariate model derived from the initial model, set at a 0.05 significance level.

**Table 3:** Initial and final multiple Poisson regression models to identify factors associated with anemia in children aged six to 59 months. Recife (PE), Brazil.

Variables	Initial model		Final model	
	PR (95%CI)	p	PR (95%CI)	p
Maternal age (years)		0.812		
<20	1.21 (0.66-2.23)	0.533		
20 to 35	1.06 (0.67-1.69)	0.790		
≥36	1.0 (Ref.)			
Per capita income (MW)		0.877		
<0.5	0.80 (0.13-4.93)	0.809		
≥0.5 and <1.0	0.70 (0.11-4.62)	0.710		
≥1.0	1.0 (Ref.)			
Maternal hemoglobin		0.035		0.019
<12 g/dL	1.49 (1.03-2.16)	0.035	1.53 (1.07-2.19)	
≥12 g/dL	1.0 (Ref.)		1.0 (Ref.)	
Child’s age (months)		0.055		0.035
6 to 24	1.41 (0.99-2.01)	0.055	1.44 (1.03-2.03)	
25 to 60	1.0 (Ref.)		1.0 (Ref.)	
Diarrhea in the previous two weeks		0.010		0.006
Yes	1.64 (1.13-2.38)	0.010	1.67 (1.16-2.41)	
No	1.0 (Ref.)		1.0 (Ref.)	
Weight-for-age		0.056		0.014
Underweight/nutritional risk	1.98 (1.07-3.66)	0.030	2.26 (1.30-3.93)	0.004
Nutritional adequacy	1.77 (1.08-2.90)	0.025	1.76 (1.09-2.85)	0.020
Overweight/obesity	1.0 (Ref.)			
Length/height-for-age		0.158		
Stunted	1.42 (0.87-2.30)	0.158		
Normal length/height	1.0 (Ref.)		
EBF:		0.054		0.064
<6 months	1.69 (0.99-2.87)	0.054	1.67 (0.97-2.87)	0.064
≥6 months	1.0 (Ref.)		1.0 (Ref.)	

* PR: prevalence ratio; 95%CI: 95% confidence interval; MW: minimum wage; EBF: exclusive breastfeeding.

## DISCUSSION

The 1997/2006/2016 historical series unquestionably confirmed the remarkable and even surprising reduction in the prevalence of anemia in children under five years in Pernambuco, dropping from 40.9 to 24.2%, that is, a 40% decrease in less than 20 years. Surely, such a reduction, which would be impressive in any country, means that risk factors have been mitigated or disappeared, which should be reflected in the factorial models analyzed by multivariate statistical tests.

In Brazil (Maceió, Alagoas), a study published in 2017 evaluated the evolution of anemia in children based on two studies conducted in 2005 and 2015, revealing a clear downward trend in this population — from 45.1 to 27.4%^22^. As in Pernambuco, this drop can be mainly attributed to similar conditions: fortification of highly consumed food products (industrialized wheat and corn foods) with iron and folate^23^. In the history of other countries, food fortification with antianemic agents was also a strategically important measure to fix the problem. In a way, this resource has been incorporated into food and nutrition public policies, either as a preventive or generic curative action, specifically aimed at more vulnerable strata of the population, such as children and pregnant women.

By adopting food fortification in 2004, Brazil pragmatically and effectively advanced in the fight against anemias, with more comprehensive, integrated, well-designed, and successful policies, resulting in the great achievement of leaving the hunger map^24,25^. Related actions, such as the Bolsa Família Program, the increase in the minimum wage, and the improvement in school meals, led to a substantial reduction in the level of childhood anemia in Pernambuco.

This study, which evaluates the evolution of anemia by analyzing three nutritional surveys in Pernambuco covering the last years of the previous century and the first decade of the 2000s, reveals an initiative that should be promoted and improved. In this regard, 2004 is a historic milestone. The experience learned must be expanded and renewed, following not only the lessons from other countries but especially our own.

The historical trend might not be confirmed, as the low prevalence of anemias in children is a new fact in the latest epidemiological scenario. In other words: the decreased prevalence of anemia among children in Pernambuco (equivalent to 40% between PENS II and IV) implies (and is explained by) the progressive elimination of risk factors. This is the case for groups of variables such as household income, cough, hospitalization in the previous 12 months, and other categories.

The formal logic of possible relationships is denied by the material logic of current standard data. Many variables validated in past surveys of the same population in the same place may lose their validity to new historically changed conditions. When comparing data from 1997 (PESN II) and 2016 (final year of the historical series), the prevalence of anemia among children in Pernambuco had a significant decrease: 40%. Yet, at the same time, the most recent risk factors have undergone a major change. It is the dynamics of facts. Actually, this change occurred in the first 10 years of this century, when food and nutrition problems gained prominence and became high priorities in Brazilian public policies.

We underline that anemia in children was significantly associated with anemia in mothers, which should be considered during prenatal care. Starting the treatment for iron-deficiency anemia in pregnant women as early as possible is essential to optimize its protective effects on the fetus^26^.

Children under 24 months has a higher prevalence of anemia (37.7%) compared to those older than 24 months (21.2%). This statistically significant difference between prevalence rates might result from the accelerated growth and consequent increase in the nutritional needs of younger children, in addition to early weaning and a diet poor in nutrients and often very monotonous, derived from a previous predominantly dairy diet^27^. A similar situation was found in a study conducted in Gondar, Ethiopia, which identified a higher prevalence of anemia in children under 11 months (48.9%) compared to the age group of 48 to 59 months (8.9%)^5^. In Peru, a higher prevalence of anemia was also detected in children aged six to 12 months, 87.3%, contrasting with 58.7% in the age group of 19 to 36 months^28^.

The report of diarrhea in the two weeks prior to the interview was associated with anemia in children, both when assessed separately in the bivariate analysis and together with other determinants in the multivariate model. This association also occurred in the 1997 PESN^18^, but not in 2006^19^, as diarrhea was not selected for the final model in this year. The predisposition to anemia after an episode of acute infection, such as diarrhea, is evident, but its effects depend on the severity and duration of the process in order to become a detectable risk factor for anemia^29^.

The association identified in this study between anemia and nutritional deficiency, demonstrated by the W/A and L/A or H/A indices, is also described in PESN III^19^, whose indices were statistically significant compared to anemia in both investigations. This finding corroborates a study carried out in Bangladesh, which revealed that malnourished children had a higher prevalence of anemia^30^, and another from Nepal, where underweight children were more likely to have moderate to severe anemia^31^.

The lack of information on the food consumption of children under five years is the main limitation to the interpretation of our results. The nutritional value of the diet is crucial when analyzing the problem of anemia, since its most common cause in children is iron deficiency^1^, usually associated with insufficient intake of this micronutrient or the consumption of foods that inhibit its absorption in the digestive tract^27^.

We highlight that between 1997 and 2016, the prevalence of anemia gradually decreased in children aged six to 59 months in Pernambuco, evidencing a downward epidemiological trend. Nonetheless, our most expressive and even historical contribution was revealing the remarkable and consistent evidence that anemia has a new perspective among children in Pernambuco, the Northeast Region, and possibly Brazil, by dropping from 40.9 to 24.2% in less than 20 years.

Publications showing that anemia, as well as vitamin A deficiency, in children^25^ and pregnant women has been decreasing in a seemingly consistent way justify the organization of a forum to discuss and forward these problems at the national or, at least, regional level, so as to obtain technical and even political-administrative positions on these issues, which should not be limited to the purely academic domain of dissertations and theses.
